# Transcutaneous electrical acupoint stimulation for postoperative cognitive dysfunction in geriatric patients with gastrointestinal tumor: a randomized controlled trial

**DOI:** 10.1186/s13063-021-05534-9

**Published:** 2021-08-23

**Authors:** Lijuan Xi, Fang Fang, Haijuan Yuan, Daorong Wang

**Affiliations:** 1 School of Nursing, Yangzhou University, Yangzhou, 225009 Jiangsu China; 2 Clinical Medical College of Yangzhou University, Yangzhou, 225001 Jiangsu China; 3 General Surgery Institute of Yangzhou, Yangzhou University, Jiangsu, Yangzhou, 225001 China; 4 Department of Gastrointestinal Surgery, Northern Jiangsu People’s Hospital, No.98 Nantong West Road, Yangzhou, 225001 Jiangsu China

**Keywords:** POCD, TEAS, Gastrointestinal tumor

## Abstract

**Background:**

This study aimed to evaluate the effect of perioperative transcutaneous electrical acupoint stimulation (TEAS) on postoperative cognitive dysfunction (POCD) in older patients who were diagnosed with gastrointestinal tumor and received radical resection of gastrointestinal tumors under general anesthesia.

**Methods:**

A total of 68 patients who received radical resection of gastrointestinal tumors under general anesthesia were randomly divided into two groups. TEAS group patients received TEAS treatment. The treatment time was 30 min before the induction of anesthesia until the end of the surgery, 1 day before operation and from the first day to the third day after the operation. Except on the day of surgery, we treated the patients for 30 min once a day. In the sham TEAS group, the electronic stimulation was not applied and the treatment was the same as the TEAS group. The primary outcome was perioperative cognition evaluated by the Mini-Mental State Examination (MMSE) and secondary outcomes were the perioperative level of interleukin-6 (IL-6), S100 calcium-binding protein β (S100β), and C-reactive protein (CRP).

**Results:**

The postoperative score of MMSE, orientation, memory, and short-term recall in the sham TEAS group was significantly lower than the preoperative and TEAS group (*P* < 0.05). The incidence of POCD in the TEAS group (21.88%) was lower than those in the sham TEAS group (40.63%). S100β, IL-6, and CRP in the TEAS group were significantly lower than those in the sham TEAS group on the third day after the operation (*P*< 0.05). Postoperative S100β, IL-6, and CRP in two groups were significantly higher than those before operation except for S100β on the third day after the operation in the TEAS group (*P* < 0.05).

**Conclusions:**

Perioperative TEAS treatment reduced the postoperative inflammatory response and increased the postoperative cognitive function score and decrease the incidence of POCD in geriatric patients with gastrointestinal tumor.

**Trial registration:**

ClinicalTrials.gov NCT04606888. Registered on 27 October 2020. https://register.clinicaltrials.gov.

## Background

Postoperative cognitive dysfunction (POCD) is one of the common complications of central nervous system in cancer patients with a 8.9–46.1% incidence [1, 2]. It is mainly manifested as the decline or damage of attention, memory, perception, abstract thinking, executive, language response, body movement, and other functions [3, 4]. It is not easy to identify, but it can last for months, years or even become a permanent disease [5], which can severely affect patients’ postoperative recovery, prolong the hospitalization time, increase the medical cost, affect the social function of patients, reduce the quality of life, and increase the mortality [6].

Surgical stress and inflammation are contributing factors for the development of POCD [7]. Surgical trauma can induce a systematic inflammatory response and release systematic inflammatory mediators, such as CRP, IL-6, which can enter the central nervous system (CNS) via the relatively permeable blood-brain barrier (BBB) and activate microglial cells to secrete additional cytokines and lead to CNS inflammatory [1]. In order to prevent the development of POCD among older patients, the discovery of effective interventions reducing inflammatory response is important. The treatment of parecoxib sodium [8], dexmedetomidine [9], and urinastatin [10] in the perioperative period can reduce inflammatory reaction and increase postoperative cognitive function. However, these drugs have side effects, such as neutropenia, diarrhea, skin redness, and other adverse reactions. The intervention of nerve block also makes the sensory and motor function of the innervated area temporarily lose and blocks the upload of noxious stimulation, which can inhibit the adverse stress reaction in the perioperative period and reduce the incidence of POCD [11]. But it is invasive and requires advanced requirements for anesthesia technology, and it may increase the expenditure of patients.

Transcutaneous electrical acupoint stimulation (TEAS) is a non-invasive acupoint stimulation therapy [12] which combined the preponderances of both acupuncture and transcutaneous electrical nerve stimulation (TENS). It can input low-frequency pulse current into human acupoints through electrodes pasted on the surface of skin [13]. TEAS treatment can reduce the intraoperative anesthetic consumption, decrease the incidence of postoperative nausea and vomiting (PONV) [14], and improve the postoperative recovery of patients [12]. Recently, TEAS treatment was found to improve the cognitive function of geriatric patients with silent lacunar infarction [13]. However, previous studies of TEAS treatment on cognition mainly focused on the intraoperative while the effect of perioperative TEAS treatment on POCD is not clear.

### Purpose

We aimed to evaluate the effect of perioperative TEAS on POCD in older patients who were diagnosed with gastrointestinal tumor and received radical resection of gastrointestinal tumors under general anesthesia.

## Methods

### Study design

We conducted this prospective, randomized, single-blind, intervention-controlled clinical trial in Subei People’s Hospital of Jiangsu province. This protocol was approved by the Ethics Committee of Subei People’s Hospital of Jiangsu province (2020ky-046). This article was written using the CONSORT 2010 checklist (see Additional file 1).

### Participants

Patients who volunteered for the study were selected from August 2020 to October 2020. All patients signed informed consent before this study.

The inclusion criteria are ① patients aged 60 years or older, ② patients were diagnosed with gastrointestinal tumor and received radical resection of gastrointestinal tumors under general anesthesia in Subei People’s Hospital of Jiangsu province, ③ patients understood the research content and signed the informed consent form, ④ American Society of Anesthesiology (ASA) score I–III, ⑤ no frailty before operation, and ⑥ D-dimer was normal before the operation. The exclusion criteria are ① patients with cognitive dysfunction before the operation or patients with previous history of cognitive dysfunction, dementia, and delirium; ② patients with a history of severe depression, schizophrenia, and other mental and nervous system diseases or taking antipsychotic or antidepressant drugs in the past; ③ patients with severe hearing or visual impairment due to eye or ear diseases without assistive tools; ④ patients who are unable to communicate or have difficulty communicating; ⑤ according to the definition of “China chronic disease and its risk factors monitoring report (2010)” (male average daily pure alcohol intake ≥ 61 g, female average daily pure alcohol intake ≥ 41 g, alcohol volume (g) = alcohol consumption (ML) × alcohol content% × 0.8); ⑥ patients who were hospitalized for 3 months or more before surgery or had received surgical treatment within 3 months; ⑦ patients who can’t take care of themselves or are physically disabled and unable to carry out nerve function test; ⑧ patients with severe heart, liver, and renal failure; ⑨ patients with hypoxemia (blood oxygen saturation < 94%) more than 10 min during operation; ⑩ patients admitted to ICU after operation; ⑪ patients who quit or died due to noncooperation or sudden situation; ⑫ patients who already participate in other clinical studies which may influence this study; ⑬ patient who underwent emergency surgery; and ⑭ patients who had a history of recent or conventional acupuncture treatment.

### Sample size calculation

The sample size of this research was calculated based on the following equation: *n* = (*Z*_*α*_ + *Z*_*β*_)^2^ × 2*σ*^2^/*δ*^2^. *α* is 0.05, *Z* is bilateral, and the degree of assurance (test efficiency) is 0.9. *σ* represents the average MMSE scores on the third day after operation between two groups and according to the pre-experiment, *σ* = 1.85, *δ*= 1.6. After calculation, each group needs 28 patients and expands the sample size by 20%, and the final sample size is 34 cases per group and therefore a total of 68 subjects are needed.

### Randomization and blindness

**Fig. 1:**
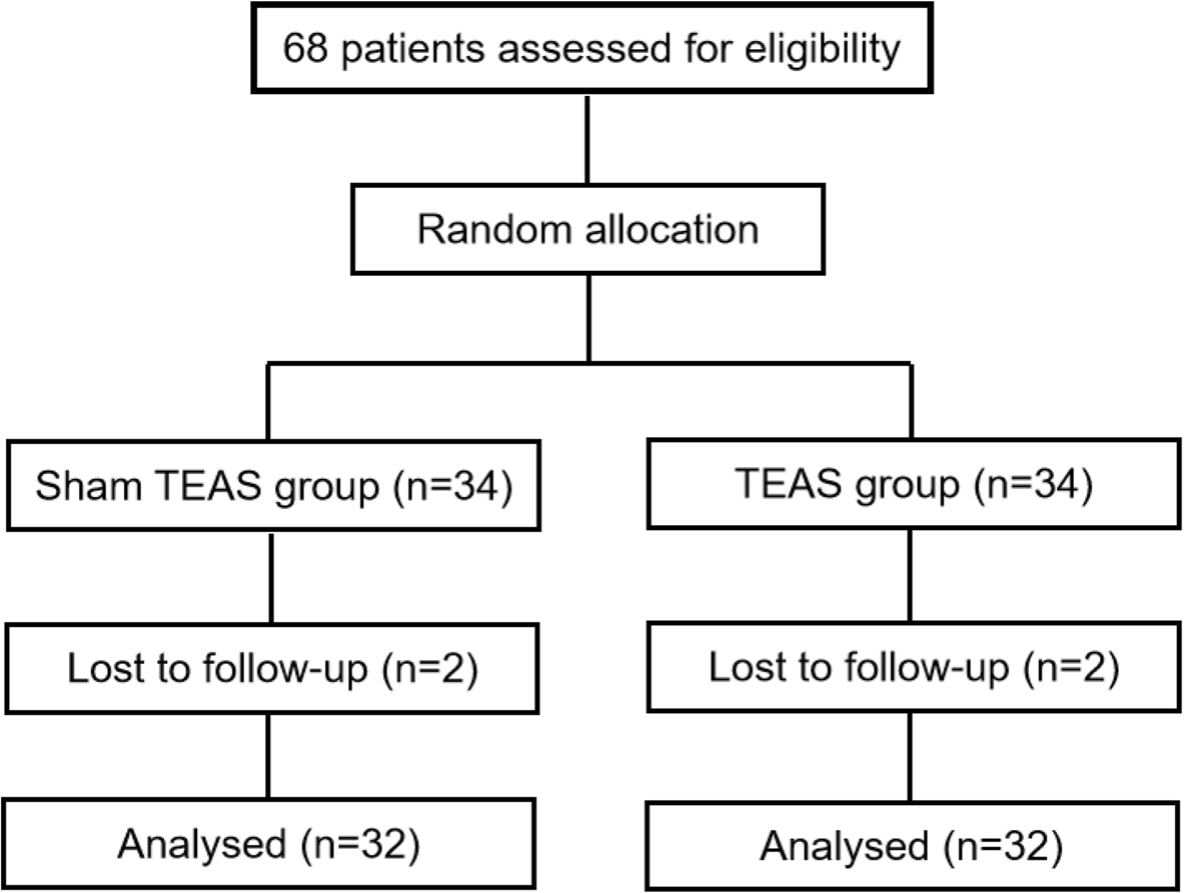
Flowchart

68 random numbers generated by SPSS 24.0 software (seed = 20160648) were put into a sealed opaque envelope for patients to choose. Patients were assigned to either the TEAS group or sham TEAS group on the basis of random numbers at the ratio of 1:1. Patients know the number but not the allocation. The randomization process was completed by a study administrator not involved in the study. Due to the unique feature of TEAS, we can only blind the patients, data collectors, technicians who tested the blood index but not the interveners. The flow chart is presented in Fig. 1

### Anesthesia management

All patients were given Tracheal Intubation General Anesthesia without preoperative medication. After entering the operating room, the intravenous access was established, and multi-functional monitor was connected to monitor hemodynamics, electrocardiography (ECG), noninvasive blood pressure, blood oxygen saturation, and heart rate. Anesthesia induction includes the following: midazolam 0.04–0.06 mg/kg, sufentanil 0.5–1 μg/kg, and cisatracurium besilate 0.1–0.4 mg/kg. After tracheal intubation, mechanical ventilation was performed. The parameters were tidal volume of 8–10 ml/kg, respiratory rate 12–16 times/min, the ratio of inhalation to exhalation 1:2, and the oxygen flow rate 2 L/min to maintain the end-tidal carbon dioxide partial pressure (PECO2) at 30–45 mmHg. During the operation, continuous oxygen was given at 2 L/min, sevoflurane 1.0–1.5%, sufentanil 0.2 μg/(kg h), dexmedetomidine 0.2–0.4 μg/(kg h), and cisatracurium besilate for injection 0.06–0.12 mg/(kg h) and propofol 6–9 g (kg h) were used to maintain the bispectral index (BIS) at 40–60. All patients were given sufentanil combined with dezocine for patient-controlled intravenous analgesia (PCIA). PCIA configuration includes the following: sufentanil 5–15 μg/kg + 0.1–0.4 mg/kg dezocine.

### Intervention

**Fig. 2:**
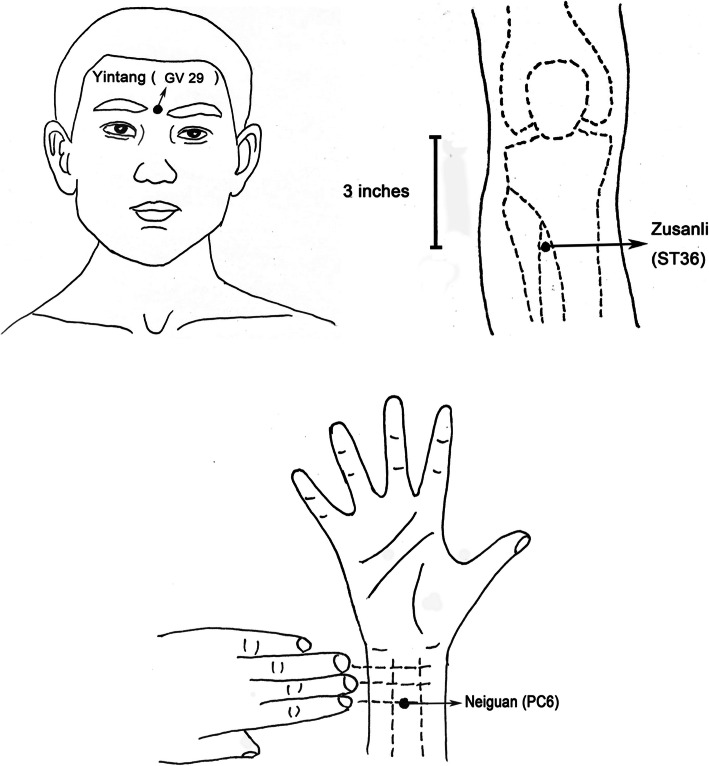
Location of acupoints

According to the theory of “Xingnao Kaiqiao Acupuncture” in traditional Chinese medicine [15], three acupuncture points were selected as the target points: bilateral Neiguan [16] (PC6, on the palmar side of the forearm, 3 cm proximal to the transverse carpal crease, between the palmaris longus tendon and the radial flexor carpi tendon), Yintang [17] (GV29 ], on the forehead of the human body, between the two eyebrows) and bilateral Zusanli [18] (ST36, 3 inches below the outer knee). Patients in the TEAS group received perioperative TEAS by two experienced researchers and the treatment time was 30 min before the induction of anesthesia until the end of the surgery, 1 day before operation, and from the first day to the third day after the operation. Except on the day of surgery, we treated the patients 30 min once a day. We used transcutaneous electrical stimulators (SDZ-III, Suzhou medical technology, Suzhou, China) to provide an altered frequency 2/100 Hz, disperse-dense waves, and adjusted intensity which was less than 10 mA. In the sham TEAS group, the electrodes were placed at the same acupoints as the TEAS group, but the electronic stimulation was not applied and they were told that they might not feel electrical stimulation (Fig. 2). To deliver the intervention, the researchers need to learn the function and positioning of acupoints, the inspection and use of an instrument, and clinical practice in the traditional Chinese Medicine Department of the hospital for 7 times, 1 h each time. And only passing the final assessment of traditional Chinese medicine can researchers begin this intervention.

### Outcomes

#### Primary outcome

The primary outcome of this study was the change in cognition in the morning of the day before operation and three days after the operation. Mini-Mental State Examination (MMSE) was used to evaluate the cognition by experienced researchers who were trained in neuropsychological assessment. The MMSE is a 30-point questionnaire used to measure orientation (time and place), memory (immediate and short term), attention, calculation, and language (naming, repetition, listening, reading, and writing) [19, 20]. Higher score means better cognitive function. According to the educational level of the subjects, the scores of illiterate < 17, primary school < 20, middle school, and above < 24 were defined as cognitive impairment. The score decreased by 2 points after the operation is considered to be POCD.

#### Secondary outcomes

Interleukin-6 (IL-6), S100 calcium-binding proteinβ (S100β), and C-reactive protein (CRP) were recorded before the operation, the 1st and 3rd day after the operation. Four milliliters blood sample was taken each time and the blood samples were immediately centrifuged at 3000 rpm for 10 min to collect serum and were stored in a freezer at − 80 °C until further assayed. The concentrations of IL-6 and S100β in serum were quantified with a commercial ELISA kit (Beyotime, China) and each blood sample was repeated 3 times. The C-reactive protein (CRP) was extracted from the case data.

#### Statistical analyses

All research data were analyzed by IBM SPSS software 24.0. Research data of normal distribution were described as the mean±SD and the comparison between the two groups was performed by independent sample *t* test. Non-normal distribution was described as the median (interquartile), and the comparison between the two groups was performed by non-parametric test. Categorical variables were described as frequency (*f*) and numbers (%) and the comparison between the two groups was performed by chi-squared test and Fisher’s exact test.

## Results

A total of 68 patients were enrolled in the study; 4 (5.9%) dropped out because they declined to undergo this procedure and 64 patients’ records were analyzed.

### General information of patients

Smoking index is indicated by the number of cigarettes per day multiply years of smoking. No significant differences were observed between these two groups in general information (*P* > 0.05, Table 1).

**Table 1:** General information of two groups of patients (*n* = 64)

Characteristics	TEAS group (*n* = 32)	Sham TEAS group (*n* = 32)	*P*
Age	69.28±7.04	72.06±6.91	0.116^a^
Gender (man/woman), *n*	22/10	24/8	0.578^b^
BMI	21.73±3.28	23.02±2.98	0.103^a^
Education level (years)	6.41±3.87	6.63±3.60	0.816^a^
Mode of residence			0.637^c^
Living alone	3	2	
Living with spouse	14	18	
Living with children	15	12	
History of surgery, *n* (0/1/2)	14/14/4	19/9/4	0.416^c^
Preoperative drugs(0/1/2/3/4/5), *n*	12/12/6/1/1/0	13/11/3/0/3/2	0.481^c^
Smoking index level, *n*			0.968^c^
Smoking index = 0	11	12	
0 < smoking index < 200	10	8	
200 ≤ smoking index ≤ 400	8	8	
Smoking index > 400	3	4	
Drinking, *n* (yes/no)	18/14	16/16	0.616^b^
Exercise time per week (h)			0.791^c^
0–3.4	19	17	
3.5–6.9	6	6	
7–10.4	5	8	
≥ 10.5	2	1	
Preoperative frailty	4.09±2.91	4.28±2.04	0.766^a^
ASA (II/III), *n*	13/19	18/14	0.211^b^
Operation type (1/2/3/4/5/6), *n*	12/7/0/3/0/10	12/12/8/2/5/1/4	0.306^c^
Operation duration	2.67±0.78	2.65±0.76	0.916^a^
Anesthesia duration	3.33±0.99	3.22±0.90	0.667^a^

Notes: ^a^independent sample *t* test; ^b^chi-squared test; ^c^Fisher’s exact test

Abbreviations: Operation type: 1 = Radical gastrectomy for gastric cancer; 2 = Laparoscopic radical gastrectomy for gastric cancer; 3 = Radical resection of colon cancer; 4 = Laparoscopic radical resection of colon cancer; 5 = Radical resection of rectal cancer; 6 = Laparoscopic radical resection of rectal cancer

### Perioperative cognition

**Fig. 3:**
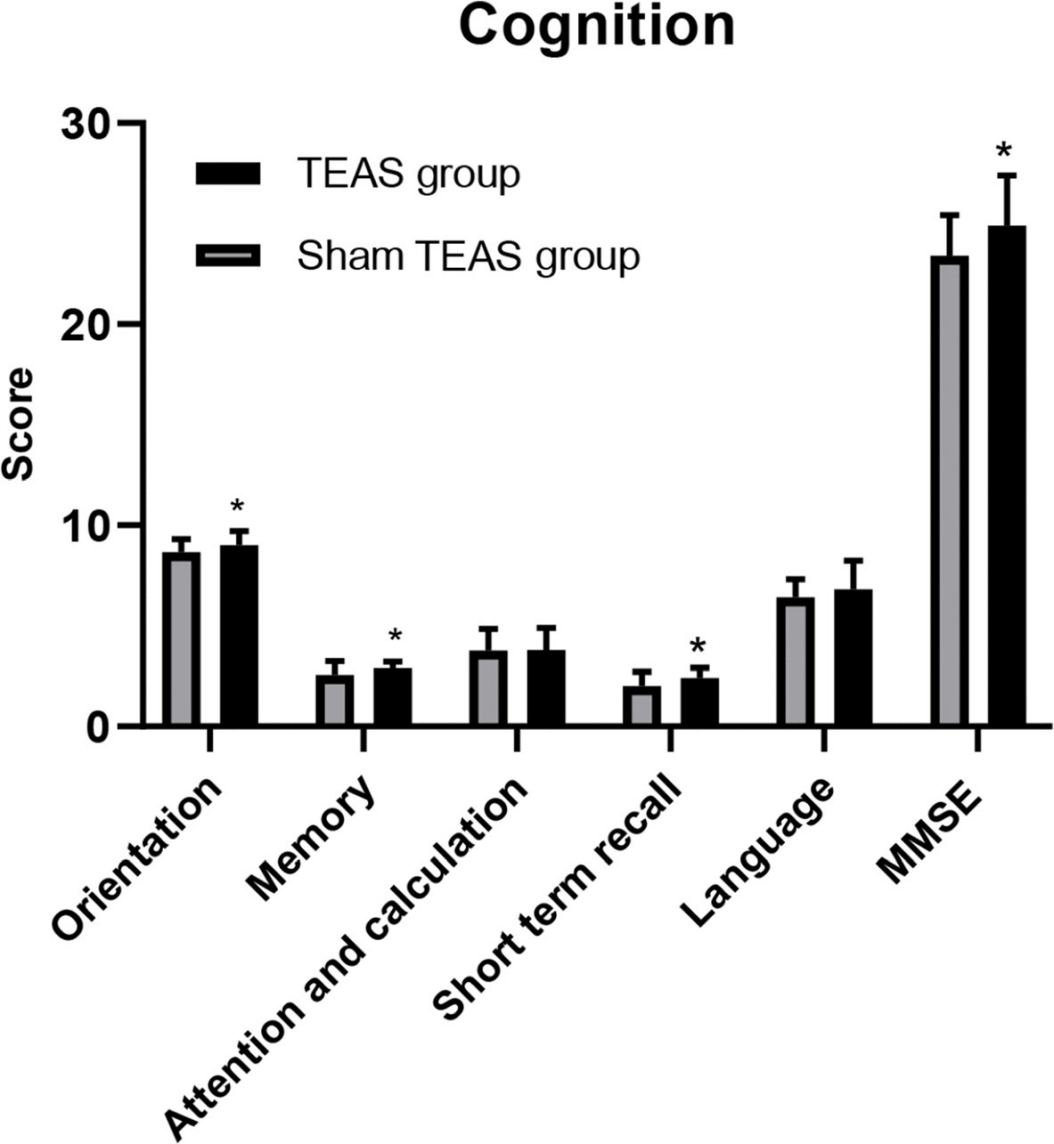
Postoperative cognition score between two groups. Note: *P < 0.05: compared with the sham TEAS group

On the third day after the operation, the total score of MMSE, and the score of orientation, memory, and short-term recall in the sham TEAS group were significantly lower than those in preoperative and TEAS groups (*P* < 0.05, Table 2, Fig. 3). The incidence of POCD in the TEAS group was 21.88%, which was lower than that in the sham TEAS group (40.63%).

**Table 2:** Perioperative cognition of two groups of patients (*n* = 64)

Indexes	Groups	Preoperative	The third day after the operation
MMSE	TEAS group	24.69±1.87	24.88±2.52*
	Sham TEAS group	25.22±1.81	23.38±2.03^#^
Orientation	TEAS group	9.16±0.68	9.00±0.72*
	Sham TEAS group	9.13±0.71	8.66±0.65^#^
Memory	TEAS group	2.91±0.30	2.88±0.34*
	Sham TEAS group	2.88±0.34	2.56±0.67^#^
Attention and calculation	TEAS group	3.81±1.18	3.78±1.10
	Sham TEAS group	4.00±1.05	3.75±1.08
Short term recall	TEAS group	2.19±0.47	2.41±0.50*
	Sham TEAS group	2.38±0.49	2.06±0.72^#^
Language	TEAS group	6.63±1.34	6.81±1.42
	Sham TEAS group	6.84±1.37	6.41±0.91
Incidence of POCD	TEAS group	-	21.88%
	Sham TEAS group	-	40.63%

Notes: Compared with preoperative, ^#^*P* < 0.05; compared with the sham TEAS group, *P < 0.05

### S100β, IL-6, and CRP Levels

**Fig. 4:**
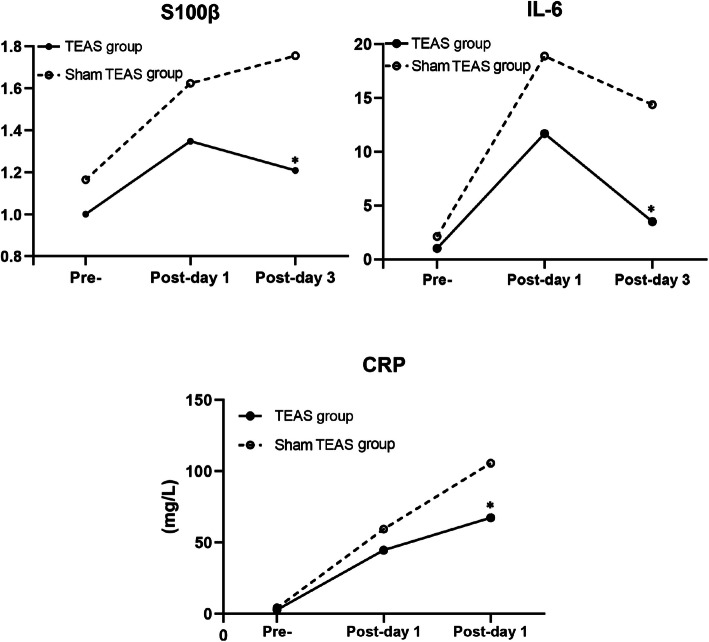
Perioperative S100β, IL-6, and CRP Levels. Note: Preoperative day (Pre); Postoperative (Post-). *P < 0.05: compared with the sham TEAS group

There were 4 missing values of CRP before the operation, 2 in the TEAS group and 2 in the sham TEAS group. We replaced the missing value with sequence average of 3.34. S100β, IL-6, and CRP in the TEAS group were significantly lower than those in the sham TEAS group on the third day after the operation. S100β, IL-6, and CRP in two groups were all significantly higher than those before operation (*P* < 0.05) except for S100β on the third day after operation in the TEAS group (Fig. 4, Table 3).

**Table 3:** Perioperative S100β, IL-6, and CRP Levels of two groups of patients

Indexes	Groups	Preoperative	The first day after the operation	The third day after the operation
S100β	TEAS group	1.00±0.59	1.35±0.66^#^	1.21±0.46^*^
	Sham TEAS group	1.16±0.60	1.62±1.01^#^	1.76±1.08^#^
IL-6	TEAS group	0.77 (0.63,1.241)	3.56 (1.86,10.84)^#^	1.63 (1.22,2.78)^#*^
	Sham TEAS group	1.09 (0.81,2.160)	5.14 (2.99,18.81)^#^	2.89 (1.48,9.98)^#^
CRP (mg/L)	TEAS group	1.20 (0.89,2.47)	44.53±24.90^#^	67.30±40.39^#*^
	Sham TEAS group	1.85 (0.99,3.49)	59.33±36.73^#^	105.57±53.18^#^

Notes: Compared with preoperative, ^#^*P* < 0.05; compared with the sham TEAS group, *P < 0.05

### Safety analysis

Any treatment-related adverse events (AEs) were monitored and documented throughout the trial within 24 h after each treatment by the direct observation of researchers and the self-report of the patients. Potential AEs of TEAS used in the trial include continuous post-electrostimulation sensation and skin numbness; allergic reaction; fainting; vomiting; redness, swelling, pain, or other injury on the skin during acupuncture treatment. And no AEs were reported in either group during the clinical trial.

## Discussion

In this study, we preliminary explored the effect of perioperative TEAS treatment on the values of S100β, IL-6, CRP, and the incidence of POCD.

Many studies have shown the beneficial effect of TEAS [21, 22]. However, the selection of acupoints in each study is different. According to the theory of “Xingnao Kaiqiao Acupuncture” [15] in traditional Chinese medicine, we chose the bilateral Neiguan (PC6), Yintang (GV 29), and bilateral Zusanli (ST36) as the target points. PC6 belongs to the acupoints of the pericardium meridian of hand Jueyin. Pericardial meridian is related to people’s mental activities and thinking consciousness and closely related to the function of the brain. It is the first choice for brain injury, which has functions of calming panic, relieving palpitation, nourishing heart and mind, broadening chest, and regulating Qi [23]. Acupuncture on PC6 can promote the balance of oxygen supply and demand of brain cells, improve the blood circulation of brain tissue, and finally improve the cognition of dementia patients [24]. GV 29 is an important acupoint of the governor vessel, which has the function of refreshing the brain and activating collaterals [24]. The electro-acupuncture at GV 29 can improve postoperative cognition [25]. ST36 is one of the main acupoints in the stomach meridian of Foot Yangming. It has the functions of regulating the spleen and stomach, tonifying the middle, replenishing Qi, dredging meridians, activating collaterals, dispersing weathering and dampness, strengthening the body, and eliminating pathogenic factors [26]. Acupuncture at ST36 can activate most parts of the brain, especially in the temporal lobe, so as to regulate body movement, sensation, language, learning and memory, mental emotion, and internal organs activities [26].

MMSE is one of the most influential, popular, and commonly used cognition screening scale in the world [27, 28]. Our study showed that perioperative TEAS can improve the total score of MMSE and decrease the incidence rate of POCD on the third day after operation. Perioperative treatment of TEAS on cognition is relatively rare, and some studies have shown that TEAS treatment from 30 min before anesthesia to the end of surgery can reduce the incidence of POCD in patients undergoing radical thoracoscopic lung cancer operation and gynecological laparoscopy surgery [28]. Also, our study first showed that the TEAS treatment can increase three dimensions score of orientation, memory, and short-term recall of the cognition, and this finding needs more study to conform.

S100β is a biomarker involving in the mechanism of cognitive impairment, which existed in several types of cell in the peripheral and central nervous system [CNS] [29, 30]. IL-6 is a pro-inflammatory cytokine which can mediate inflammatory and immune responses in CNS [31]. CRP is a non-specific biomarker of systemic bodily inflammation [32, 33] which can accelerate the development of neurodegenerative disorders through activating microglia, increasing levels of proinflammatory cytokines, and activating the complement cascade [34, 35]. In this study, the value of S100β and IL-6 was detected twice. Therefore, in order to avoid the influence of experimental error on the experiment, the method of averaging was adopted to evaluate the level of S100β and IL-6. Our study showed that perioperative TEAS treatment decreased the postoperative S100β, IL-6, and CRP which was consistent with other studies [13, 36, 37]. However, these three inflammatory cytokines have no difference between the two groups on the first day after the operation [38, 39]. And the reason for this result may be due to the small sample size, so more studies are needed to confirm this result in the future.

Owning to the situation that previous studies of the TEAS treatments on cognition mainly focused on intraoperative and the effect of perioperative TEAS on POCD is not clear, we investigated the effect of perioperative TEAS treatment on POCD. However our study had its limitations, which were as follows: we just implemented the perioperative TEAS treatment in geriatric patients with gastrointestinal tumor, whether this treatment is effective on other diseases needs more studies to be identified. Furthermore, the cognition was measured only at the third day after the operation and long-term follow-ups such as postoperative 7 days, 1 month, and 1 year may be different.

## Conclusion

In summary, perioperative TEAS treatment can reduce the postoperative inflammatory response and increase the postoperative cognitive function score and decrease the incidence of POCD in geriatric patients with gastrointestinal tumor.

## Data Availability

Patients’ demographic information including mode of residence, education level, sports situation, smoking index, drinking, were asked by the trained researchers to the patients. Age, gender, Body Mass Index (BMI), history of surgery, preoperative drugs, American Society of Anesthesiologists (ASA), operation type, operation duration, and anesthesia duration were all extracted from medical records. Perioperative IL-6 levels were also extracted from medical records. The measure of S100β and CRP was repeated 3 times. MMSE were proved to have great reliability and validity.

## Abbreviations

TEAS: Transcutaneous electrical acupoint stimulation
POCD: postoperative cognitive dysfunction
MMSE: Mini Mental State Examination
CRP: C-reactive protein
IL-6: Interleukin-6
S100β: S100 calcium-binding protein β
AEs: Adverse events

## References

[1] Kristek G, Radoš I, Kristek D, Kapural L, Nešković N, Škiljić S, et al. Influence of postoperative analgesia on systemic inflammatory response and postoperative cognitive dysfunction after femoral fractures surgery: a randomized controlled trial. Reg Anesth Pain Med. 2019;44(1):59–68. 10.1136/rapm-2018-000023. 10.1136/rapm-2018-000023 30640654

[2] Li Y, Huang D, Su D, Chen J, Yang L (2019). Postoperative cognitive dysfunction after robot-assisted radical cystectomy (RARC) with cerebral oxygen monitoring an observational prospective cohort pilot study. BMC Anesthesiol.

[3] Rasmussen L, Johnson T, Kuipers H, Kristensen D, Siersma V, Vila P, Jolles J, Papaioannou A, Abildstrom H, Silverstein JH, Bonal JA, Raeder J, Nielsen IK, Korttila K, Munoz L, Dodds C, Hanning CD, Moller JT, ISPOCD2(International Study of Postoperative Cognitive Dysfunction) Investigators (2003). Does anaesthesia cause postoperative cognitive dysfunction? A randomised study of regional versus general anaesthesia in 438 elderly patients. Acta Anaesthesiol Scand.

[4] Amanzio M, Palermo S, Zucca M, Rosato R, Rubino E, Leotta D, Bartoli M, Rainero I (2018). Neuropsychological correlates of instrumental activities of daily living in neurocognitive disorders: a possible role for executive dysfunction and mood changes. Int Psychogeriatr.

[5] Evered L, Silbert B (2018). Postoperative Cognitive Dysfunction and Noncardiac Surgery. Anesth Analg.

[6] Borges J, Moreira J, Moreira A, Santos A, Abelha F (2017). Impact of postoperative cognitive decline in quality of life: a prospective study. Rev Bras Anestesiol.

[7] Kotekar N, Shenkar A, Nagaraj R (2018). Postoperative cognitive dysfunction - current preventive strategies. Clin Interv Aging.

[8] Huang J, Lv Z, Zhang B, Jiang W, Nie M (2020). Intravenous parecoxib for early postoperative cognitive dysfunction in elderly patients: evidence from a meta-analysis. Expert Rev Clin Pharmacol.

[9] Yang W, Kong L, Zhu X, Wang R, Liu Y, Chen L (2019). Effect of dexmedetomidine on postoperative cognitive dysfunction and inflammation in patients after general anaesthesia: A PRISMA-compliant systematic review and meta-analysis. Medicine..

[10] Lv Z, Huang J, Zhang J, Zhang J, Guo J, Chen A (2016). Effect of Ulinastatin in the Treatment of Postperative Cognitive Dysfunction: Review of Current Literature. Biomed Res Int.

[11] Deng L, Hou L, Song F, Zhu H, Zhao H, Chen G (2017). Effect of pre-emptive analgesia by continuous femoral nerve block on early postoperative cognitive function following total knee arthroplasty in elderly patients. Exp Therapeutic Med.

[12] Zhang Q, Gao Z, Wang H, Ma L, Guo F, Zhong H, Xiong L, Wang Q (2014). The effect of pre-treatment with transcutaneous electrical acupoint stimulation on the quality of recovery after ambulatory breast surgery: a prospective, randomised controlled trial. Anaesthesia..

[13] Gao F, Zhang Q, Li Y, Tai Y, Xin X, Wang X, Wang Q (2018). Transcutaneous electrical acupoint stimulation for prevention of postoperative delirium in geriatric patients with silent lacunar infarction: a preliminary study. Clin Interv Aging.

[14] Liu Y, Tang W, Gong S, Chan C (2017). A Systematic Review and Meta-Analysis of Acupressure for Postoperative Gastrointestinal Symptoms among Abdominal Surgery Patients. Am J Chinese Med.

[15] Lu G, Zhang Y, Wang T, Gu Y, Lei J, Cui M, Pan XH, Ma WJ, Guo SL (2020). Assessment of efficacy of acupuncture combined with hyperbaric oxygen therapy for patients with delayed encephalopathy of CO intoxication by magnetic resonance voxel incoherent motion imaging. Acupuncture Res.

[16] Wang C, Liang X, Yu Y, Li Y, Wen X, Liu M (2020). Electroacupuncture pretreatment alleviates myocardial injury through regulating mitochondrial function. Eur J Med Res.

[17] Xi H, Wu W, Liu C, Wang X, Zhao Y, Zheng S, Li JH (2020). Effect of Tongdu Tiaoshen needling in treatment of chronic insomnia by regulating the hypothalamic-pituitary-adrenal axis. Acupuncture Res.

[18] Yang H, Yang H, Wang L, Shi H, Liu B, Lin X, Chang Q, Chen JDZ, Duan Z (2020). Transcutaneous Neuromodulation improved inflammation and sympathovagal ratio in patients with primary biliary ssscholangitis and inadequate response to Ursodeoxycholic acid: a pilot study. BMC Complement Med Ther.

[19] Terazawa S, Oshima H, Narita Y, Fujimoto K, Mutsuga M, Tokuda Y, Yoshizumi T, Ito H, Uchida W, Usui A (2018). Strategy of Cardiovascular Surgery for Patients With Dementia as Evaluated by Mini-Mental State Examination. Circ J.

[20] Folstein MF, Folstein SE, Mchugh PR (1975). Mini-mental state. J Psychiatr Res.

[21] Yu X, Zhang F, Chen B (2020). The effect of TEAS on the quality of early recovery in patients undergoing gynecological laparoscopic surgery: a prospective, randomized, placebo-controlled trial. Trials..

[22] Bai Y, Gao C, Li W, Du Y, An L (2020). Transcutaneous electrical acupuncture stimulation (TEAS) for gastrointestinal dysfunction in adults undergoing abdominal surgery: study protocol for a prospective randomized controlled trial. Trials..

[23] Tang Y, Cui X, Liu K, Li X, Han S, Zhao J, Wang SY, Chen YY, Gao XY, Zhu B (2020). Manual acupuncture stimulation of acupoints at the same and adjacent spinal segments promotes uterine contractility. Acupuncture Res.

[24] Lin S, Yin Z, Gao J, Zhou L, Chen X (2014). Effect of acupuncture-anesthetic composite anesthesia on the incidence of POCD and TNF-alpha, IL-1beta, IL-6 in elderly patients. Chinese J Integr Tradit Western Med.

[25] Yuan J, Wu Y, Li J, Chen X, Zhang L, Liu Y, Tong SX, Deng FF (2016). Effect of Dexmedetomidine Combined Electrical Stimulation on Coanitive Function of Patients Receiving Extracerebral Intervention. Chinese J Integr Tradit Western Med.

[26] Zhang C, Shi N, Ouyang G (2020). Effect of electroacupuncture on intestinal calcium absorption and expression of transmembrane calcium transport-related receptors in ovariectomized rats. Acupuncture Res.

[27] Zhang C, Shi N, Ouyang G. Effect of electroacupuncture on intestinal calcium absorption and expression of transmembrane calcium transport-related receptors in ovariectomized rats. Acupuncture research. 2020;45(9):702–7. 10.13702/j.1000-0607.190957 32959551

[28] Chua XY, Choo RWM, Ha NHL, Cheong CY, Wee SL, Yap PLK. Mapping modified Mini-Mental State Examination (MMSE) scores to dementia stages in a multi-ethnic Asian population. Int Psychogeriatr. 2019;31(1):147–51. 10.1017/S1041610218000704. 10.1017/S1041610218000704 30017004

[29] Wu H, Brown E, Acharya N, Appelt D, Marks A, Nagele R (2016). Age-dependent increase of blood-brain barrier permeability and neuron-binding autoantibodies in S100B knockout mice. Brain Res.

[30] Barha C, Hsiung G, Liu-Ambrose T (2019). The Role of S100B in Aerobic Training Efficacy in Older Adults with Mild Vascular Cognitive Impairment: Secondary Analysis of a Randomized Controlled Trial. Neuroscience..

[31] Kim Y, Lee K, Kim H (2017). Serum tumour necrosis factor-α and interleukin-6 levels in Alzheimer's disease and mild cognitive impairment. Psychogeriatrics.

[32] Fernandes BS, Steiner J, Bernstein HG, Dodd S, Pasco JA, Dean OM, Nardin P, Gonçalves CA, Berk M (2016). C-reactive protein is increased in schizophrenia but is not altered by antipsychotics: meta-analysis and implications. Mol Psychiatry.

[33] Vintimilla R, Hall J, Johnson L, O'Bryant S (2019). The relationship of CRP and cognition in cognitively normal older Mexican Americans: A cross-sectional study of the HABLE cohort. Medicine..

[34] Russo I, Barlati S, Bosetti F (2011). Effects of neuroinflammation on the regenerative capacity of brain stem cells. J Neurochem.

[35] Blyth B, Farhavar A, Gee C, Hawthorn B, He H, Nayak A (2009). Validation of serum markers for blood-brain barrier disruption in traumatic brain injury. J Neurotrauma.

[36] Guo J, Tang W, Guo F, Yang L, Wang J, Fu G, Yuan L (2018). Transcutaneous electrical acupoint stimulation on inflammatory response and intestinal permeability in perioperative period of laparoscopic intestinal surgery. Chinese Acupuncture Moxibustion.

[37] Chi Y, Zhang W, Yang F, Su F, Zhou Y (2019). Transcutaneous Electrical Acupoint Stimulation for Improving Postoperative Recovery, Reducing Stress and Inflammatory Responses in Elderly Patient Undergoing Knee Surgery. Am J Chinese Med.

[38] Yuan J, Wu Y, Li J, Zhang L, Chen X, Chen H (2015). Protection of Transcutaneous Acupoint Electrical Stimulation for Brain Injury Undergoing Intervention: a Clinical Observation. Chinese J Integr Tradit Western Med.

[39] Li H, Wu C, Yan C, Zhao S, Yang S, Liu P, Liu X, Wang M, Wang X (2019). Cardioprotective effect of transcutaneous electrical acupuncture point stimulation on perioperative elderly patients with coronary heart disease: a prospective, randomized, controlled clinical trial. Clin Interv Aging.

[40] Ruskin D, Lalloo C, Amaria K, Stinson J, Kewley E, Campbell F (2014). Assessing pain intensity in children with chronic pain: convergent and discriminant validity of the 0 to 10 numerical rating scale in clinical practice. Pain Res Manag.

